# The Predictive Role of Tooth Extractions, Oral Infections, and hs-C-Reactive Protein for Mortality in Individuals with and without Diabetes: A Prospective Cohort Study of a 12 1/2-Year Follow-Up

**DOI:** 10.1155/2017/9590740

**Published:** 2017-06-21

**Authors:** Lise Lund Håheim, Kjersti S. Rønningen, Morten Enersen, Ingar Olsen

**Affiliations:** ^1^Department of Oral Biology, Faculty of Dentistry, University of Oslo, Oslo, Norway; ^2^Department of Paediatric Research, Division for Women and Children, Oslo University Hospital, Rikshospitalet, Oslo, Norway

## Abstract

The predictive role of high-sensitivity C-reactive protein (hs-CRP), number of tooth extractions, and oral infections for mortality in people with and without diabetes is unclear. This prospective cohort study is a 12 1/2-year follow-up of the Oslo II study, a health survey in 2000. In all, 12,764 men were invited. Health information was retrieved from 6434 elderly men through questionnaire information, serum measurements, and anthropometric and blood pressure measurements. Diabetes was reported by 425 men. Distinct differences were observed in baseline characteristics in individuals with and without diabetes. In the diabetes group, age and hs-CRP were statistically significant whereas in the nondiabetes group, age, hs-CRP, number of tooth extractions, tooth extractions for infections and oral infections combined, nonfasting glucose, systolic blood pressure, total cholesterol, regular alcohol drinking, daily smoking, and level of education were independent risk factors. The number of tooth extractions <5 was inversely related whereas more extractions increased the risk. Multivariate analyses showed that hs-CRP was a significant predictor in persons with diabetes and tooth extractions and oral infections combined; the number of teeth extracted and hs-CRP were for persons without diabetes. Infection and inflammation were associated with mortality in individuals both with and without diabetes.

## 1. Introduction

Diabetes is a progressive metabolic disorder with defects in insulin secretion from the beta cells and/or insulin resistance in peripheral tissues leading to chronic hyperglycaemia representing type 1 and type 2 diabetes, respectively. Generally, patients with diabetes (types 1 and 2) are at a greater risk of developing cardiovascular disease (CVD) [1–3]. According to Saraiva and Sposito [4], about 68% of people over 65 years of age with diabetes die from some form of CVD, and CVD deaths among adults with diabetes are two to four times higher than in those without diabetes. Diabetes develops in people at different ages with type 1 most commonly among younger people and type 2 diabetes in middle to older age groups. The prevalence increases globally and nationally. Strøm et al. report in 2006 that the sales of medication for diabetes were doubled in the last ten-year period in Norway probably due to increased prevalence and intensified treatment [5]. However, recent publications indicate a slow reduction in type 2 diabetes incidence in Norway [6, 7].

Diabetes is also associated with chronic infections such as periodontal diseases as persons with diabetes appear to have a lower threshold for the development of these infections [8]. Periodontal diseases are infections of the supporting tissues of the teeth. They may start as infection of the gingiva and progress to the periodontal tissues causing destruction of tooth attachment and eventually tooth loss [9]. Tooth extractions which most often are due to infections such as marginal or apical periodontitis have been found to predict mortality in individuals with diabetes [10, 11]. In this respect, diabetes and periodontal infections have been identified as independent risk factors for all-cause mortality because systematic treatment of these infections improves glycaemic control in diabetes patients [12–15]. The consensus report of the Joint EFP/AAP Workshop on Periodontitis and Systemic Diseases provides evidence for the beneficial effect of blood glucose control by systematic treatment of periodontal infections [16].

hs-CRP is a general indicator of inflammation and infection which also includes oral infections. It is recognized as a risk factor for CVD, a major cause for mortality in elderly men. Therefore, it is relevant to assess how hs-CRP relates to specific infections such as tooth extractions and oral infections including periodontitis.

This study explores whether tooth extractions, oral infections, or hs-CRP affects mortality in people with regard to diabetes status in a 12 1/2-year follow-up of men from the Oslo II study.

## 2. Material and Methods

### 2.1. Patient Data and Exposure Variables

**Figure 1:**
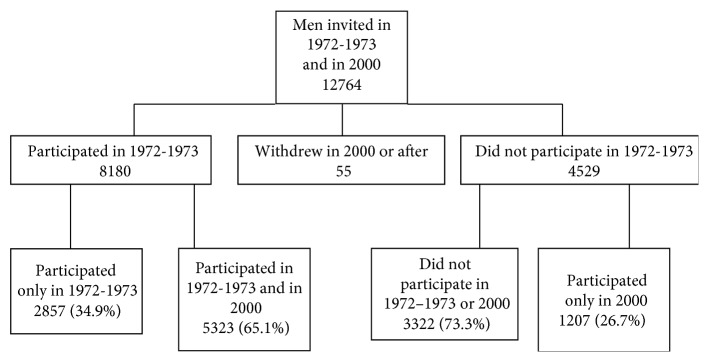
Flowchart of the Oslo study 1972/73 with the follow-up study Oslo II in 2000.

Data on diabetes and oral infections were extracted from the database of the Oslo II study [17]. The participants were men previously invited to the health screening of the Oslo study in 1972/73 (Figure 1) [17, 18]. Women were not invited in the 1972/73 screening and as a consequence not in 2000 as a major aim was to study changes in risk factors in these men. In addition, funding for including women was not available. The information comprised medical history on specific conditions including diabetes (type 1 or 2 was not specified) and dental diseases. The latter included information on tooth extraction due to periodontal infection, infection of single teeth (pulpitis), trauma, orthodontic treatment, and other causes. Number of tooth extractions for any cause was stratified into groups as follows: 1 = 0-1, 2 = 2–4, 3 = 5–8, 4 = 9–31, 5 = 32. The extraction categories were chosen to accommodate for the skewed distribution of the number of tooth extractions. Oral infections included marginal periodontitis, apical periodontitis, and nondental infections. The term oral infections in these analyses is the combined extractions due to tooth infections and any other oral infection. The other risk factors recorded and used in these analyses were smoking, alcohol drinking, insulin injections during the last 12 months, tablets to reduce blood glucose, cholesterol reducing drugs, and antihypertensiva. Blood pressure, height, weight, and waist and hip measures were recorded at the screening. Blood samples were taken and analysed for total cholesterol, HDL cholesterol, triglycerides, and nonfasting glucose. hs-CRP was measured for 5323 persons who participated in both the 1972/73 and 2000 health surveys (hence fewer numbers of fatal cases and survivors in the statistical analyses). All serum analyses were performed at the Oslo University Hospital, Ullevål, Oslo, Norway [19]. The total number of men available for analysis was 6434.

### 2.2. Endpoint Ascertainment of Total Mortality

The participants were followed with regard to cause-specific mortality from the screening in 2000 to the 31st of December 2012. Information on mortality was provided by Statistics Norway after acquiring permission from the Norwegian Data Inspectorate and the Regional Committee on Ethics in Medical Research. The study followed the Helsinki declaration. Informed consent was given by all participants that guided the future use of the screening data and the stored biological material.

### 2.3. Statistical Analyses

**Figure 2:**
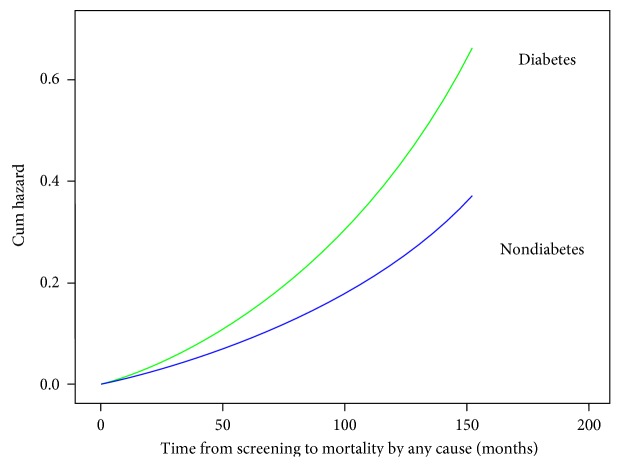
Cumulative hazard plot of the Cox proportional analysis in individuals with or without diabetes.

Descriptive statistics of included variables used in the analyses were mean with standard deviation (SD) or number and percent. hs-CRP was also analysed in ln-transformation as it normally has a skewed distribution. As no marked changes on the level of significance were observed, the variable values were not sufficiently skewed. Cox proportional hazards regression analyses were used in prediction analyses on mortality as crude, age adjusted, and in multivariate analyses. The latter analyses were adjusted for known confounders for oral infections and total mortality, namely, age, daily smoking, and level of education which indicate social status. The Cox regression plot on the hazard displays the cumulative hazard function on a linear scale (Figure 2). A *p* value <0.05 was considered significant. The IBM SPSS Statistics software program version 22 was used for the statistical analyses [20].

## 3. Results

### 3.1. Baseline Status including Oral Health in Men with respect to Diabetes Status

There were significant differences between persons with (*n* = 425) and without diabetes (*n* = 6009) for all characteristics measured at baseline (Table 1). All oral health indicators were worse in individuals with diabetes. Persons with diabetes had extracted mean 5.5 teeth (SD = 8.4) in comparison to 4.1 (6.8) in those without (*p* < 0.05). An increased level of tooth extractions due to infection was registered in individuals with diabetes (62.8%) versus those without (55.7%). Likewise, the level of periodontal infection was 12.5% versus 6.0%. The prevalence of oral infections (tooth extractions and other current oral infection combined) was 65.9% in diabetes versus 58.4%. The men with diabetes had higher nonfasting glucose, blood pressure, body mass index (BMI), serum triglycerides, and lower total cholesterol level. The level of education was slightly higher among nondiabetic men; they were younger and consumed alcohol less frequently than the diabetic counterpart. Insulin was used by 80.5% of men with diabetes and 52.2% took glucose-lowering tablets. The use of the health service differed between the two groups of men. With regard to visiting the dentist during the last year, 10.1% of men with diabetes versus 7.0% among men without diabetes attended the dentist four or more times. In contrast, 17.0% men with the disease versus 13.6% without did not attend the dentist. Four or more visits to the general practitioner were common in men with diabetes, 53.1% versus 22.2%, and no visit was reported by 8.6% of men with diabetes versus 20.1% in men without diabetes.

### 3.2. Predictors for Mortality

Cox proportional hazards analyses (Tables 2-3) showed distinct differences according to diabetes status. Age was as expected a significant predictor for mortality in the whole cohort. In age-adjusted analyses, hs-CRP (continuous scale) predicted mortality in both groups, whilst the number of tooth extractions (continuous scale), oral infections, nonfasting glucose, systolic blood pressure, total cholesterol (inversely), hs-CRP (continuous scale), alcohol-drinking pattern, daily smoking, and level of education (inversely) predicted mortality in people not having diabetes (Table 2). Total number of teeth extracted, that is, <5, was inversely related to the total mortality whereas five or more extractions increased the risk in nondiabetes. hs-CRP stratified by quartile values showed an increased risk of mortality in persons without diabetes above 1.67 mmol/l (2nd quartile value). These were statistically significant results in both diabetes and nondiabetes. Further, it was observed that the hazard ratios in diabetes for systolic blood pressure, level of education, hs-CRP, and total number of extractions were of similar sizes but not significantly different as in nondiabetes. HR of BMI was also similar but not significant.

Further multivariate Cox analyses were performed to explore independence and interaction between oral infection variables and hs-CRP (Table 3) [12]. For individuals with diabetes, age and hs-CRP (whether on a continuous scale or stratified) were independent predictors. Number of teeth extracted, oral infections, and hs-CRP were independent predictors for total mortality in individuals not having diabetes. Although not statistically significant, the hazard rate for the total number of tooth extractions were similar in diabetes and nondiabetes.

The plot on the risk of mortality over 12 1/2 years (Figure 2) shows that the cumulative hazard of having diabetes was higher than not having diabetes and increased in a curvilinear fashion with time.

## 4. Discussion

Risk factors for mortality in this 12 1/2-year follow-up study of men were distinctly different in individuals with diabetes compared to individuals without. This confirmed prior knowledge of the diabetes risk profile. The general inflammation and infection parameter hs-CRP was a significant predictor for total mortality in individuals with and without diabetes. Oral health infection factors such as the total number of extractions and number of extracted teeth for infection and oral infections combined were predictors for total mortality in individuals without diabetes. However, the hazard rate for the number of extracted teeth was similar for both groups. In a former cross-sectional analysis of this study, we found that hs-CRP was elevated in concomitant diseases and can be viewed as an indicator of the total burden of inflammations and infections in elderly persons [21]. Former analyses (ELISA) of the Oslo II study of antibodies towards antigens against oral bacteria in persons with a history of myocardial infarction versus those without showed hs-CRP not to be a predictor when adjusting for the oral infection indicating a collinearity between the two predictors, hs-CRP and oral infections [22].

Current evidence in this study indicates missing teeth as a predictor for mortality. In a 13-year follow-up of the national FINNRISK study of 1997, it was found that ≥9 missing teeth were significantly associated with mortality from CVD, diabetes, and death of any cause but not stroke [10]. In the Swedish National Study on Aging and Care in Blekinge (SNACBlekinge), 870 dentate persons (age range 60–96) were followed for 6 years [11]. The surviving dentate individuals had more teeth (mean 19.3, SD 7.9) than those who had died (mean 15.9, SD 7.3; *p* = 0.001), but periodontitis was not shown to predict mortality. Thus, there are diverging results possibly due to different modes of registration of the level of periodontitis and oral infections.

This study shows hs-CRP to be a dominant risk factor giving evidence for vulnerability in individuals with and without diabetes when exposed to infection and inflammation. hs-CRP is elevated in infectious diseases and inflammation for a number of diseases including oral infections and has been shown to be a strong independent predictor of death in people with diabetes [21, 23–27]. The current study indicates that oral infections add to the risk for total mortality both in individuals with or without diabetes. It was noticed that some of the risk factors had similar hazard ratios but were not significantly different among individuals with diabetes in contrast to individuals without diabetes. This could be due to statistical properties related to the large difference in numbers of participants in the two groups.

Metabolic syndrome that is closely linked to obesity is a major etiologic factor in type II diabetes and increased level of hs-CRP is known to be associated with obesity [28–30]. The individuals with diabetes had high blood glucose that has also been shown to increase the hs-CRP level [31]. As a consequence, the strength of hs-CRP as a risk factor for mortality in this study can be due to inflammation as well as infection. The oral microbiota has for some time been studied in relation to antibodies to oral bacteria indicating a systemic effect [9, 32]. Noncultivated bacteria identified by bacterial DNA have expanded the means for studying the systemic effect of oral infections [9]. The specific infections measured in this study are characterized by a varying but progressing disease status that can be characterized as low-grade infections with acute exacerbations [33]. The immune barrier at the gingival margin has been extensively studied [34]. Periodontal infections have also been found to contribute to an increased level of systemic infections [35, 36].

Gender differences cannot be explored in this study for reasons mentioned earlier. In this study, we wanted to elucidate the association between oral health and diabetes on mortality as we are concerned about diabetes as a complication to oral health infection and conversely oral health infections as a comorbidity in diabetes. In either cases, oral infection control is of importance to all, but in particular to persons with diabetes. Our results show that persons with diabetes have experienced significantly more tooth extractions. Judging by our results, persons with diabetes use the health service and get necessary medication that assist in the reduction of comorbidities. We have chosen to adjust for the level of education, as it is a good proximate for socioeconomic status that is considered to be related to oral health, type II diabetes, and mortality. Exploring further the association between education and these outcomes was not an aim of these analyses; however, it is of interest. Furthermore, poor health choices in general may give possible explanations for the overlap between diabetes and oral care. To achieve optimal oral health requires knowledge and means to follow-up oral health status and other health-promoting habits as physical activity and diet may play a part in diabetes.

The self-reported information on diabetes status in this study is confirmed by the basic characteristics reported by the participants. This reduces any potential selection and information bias. This is an important prerequisite for giving trust to the results on risk factors for total mortality as presented. Other previous studies have estimated the validity of specific self-reported information in health studies [37, 38]. Self-reported diabetes was over 92% reliable over time in the Atherosclerosis Risk in Communities Study [37]. The self-reported information is, however, a limitation of the current study as clinical oral measurements were not recorded. However, Buhlin et al. concluded from their study that the questionnaire information of the number of remaining teeth and use of removable dentures are reliable [38]. Less reliable is information regarding specific periodontal variables. Buhlin et al. considered, however, such data to be a valuable tool for epidemiological studies of periodontal health. To assess the direction of any reporting bias causing nondifferential misclassification of exposure can be difficult. There was also incomplete recording of oral health indicators among all participants. The strength of the current study is the reliable and complete follow-up regarding mortality by Statistics Norway that is possible due to the person identification number of Norwegian citizens.

## 5. Concluding Remarks

The results indicate that infection and inflammation are associated with mortality in both individuals with diabetes or those without. Baseline characteristics of persons with diabetes were distinctly different from person without diabetes. The general indicator for inflammation and infection, hs-CRP, and the infection variables such as the total number of tooth extractions and tooth extraction and other oral infection combined independently predicted increased risk for mortality among elderly men without diabetes. Further research is needed to get an improved understanding of the pathophysiology of tooth extractions, oral infections, and hs-CRP in diabetes and nondiabetes on total mortality.

## Figures and Tables

**Table 1:** Baseline characteristics among persons according to diabetes status. A 12 1/2-year follow-up of Oslo men of the Oslo II study of 2000.

Risk factor		Diabetes *n* = 425	Nondiabetes *n* = 6009
		Mean (SD) or *n* (%)	Mean (SD) or *n* (%)
*Oral health*			
Total number of extractions, *n* mean (SD)		5.46 (8.36)	4.11 (6.78)^∗^
Total number of tooth extractions by group, *n*, test of trend	1 = 0-1	179 (42.1)	2747 (45.7)^∗^
	2 = 2–4	111 (26.1)	1815 (30.2)
	3 = 5–8	58 (13.6)	731 (12.2)
	4 = 9–31	60 (14.1)	572 (9.5)
	5 = 32	17 (4.0)	144 (2.4)
Tooth extraction by infection (TE), *n* (%)		267 (62.8)	3350^∗^ (55.7)
Periodontitis (PI), *n* (%)		49 (12.5)	342^∗^ (6.0)
TE and PI combined (OI), *n* (%)		280 (65.9)	3510^∗^ (58.4)
*Medication*			
Antihypertensive drugs, *n* (%)		196 (54.3)	1507^∗∗∗^ (28.0)
Cholesterol-reducing drugs, *n* (%)		113 (33.0)	899^∗∗∗^ (17.0)
Take insulin, *n* (%)		342 (80.5)	—
Glucose-reducing drugs, *n* (%)		222 (52.2)	—
*General measurements*			
Age in 2000, years mean (SD)		70.4 (4.7)	69.2^∗^ (6.3)
Glucose, nonfasting, mmol/l mean (SD)		9.72 (4.0)	5.6^∗^ (1.2)
Systolic blood pressure, mmHg mean (SD)		149.7 (22.6)	144.0^∗^ (20.2)
BMI, Kg/mm^2^ mean (SD)		27.8 (3.8)	26.2^∗^ (3.3)
Total cholesterol, mmol/l mean (SD)		5.4 (1.1)	6.0^∗^ (1.1)
Triglycerides, mmol/l mean (SD)		2.3 (1.3)	1.9^∗^ (1.1)
HDL, mmol/l mean (SD)		1.3 (0.4)	1.4^∗^ (0.4)
hs-CRP, mmol/l mean (SD) *n* diabetes 331, nondiabetes 4662		4.5 (9.3)	3.5^∗^ (9.0)
Alcohol drinking, 4–7 times per week, *n* (%)		309 (72.7)	3792 ^∗^ (63.1)
Daily smoking, *n* (%)		77 (18.1)	1247 (20.8)
Level of education, 1–12 mean (SD)		11.7 (3.6)	12.2^∗^ (3.4)
*Health service use*			
Visit to the dentist in the last 12 months	None	68 (17.1)	796 (13.6)^∗∗^
	1–3 times	290 (72.9)	4630 (79.4)
	4 or more times	40 (10.1)	406 (7.0)
Visit to a general practitioner in the last 12 months	None	35 (8.6)	1175 (20.1) ^∗∗∗^
	1–3 times	155 (38.3)	3389 (57.9)
	4 or more times	215 (53.1)	1286 (22.2)

^∗^ *p* < 0.05; ^∗∗^*p* < 0.01; ^∗∗∗^*p* < 0.001.

**Table 2:** Age-adjusted univariate Cox analyses for total mortality—individuals with or without diabetes. A 12 1/2-year follow-up of the Oslo II study of 2000.

Risk factors	Total mortality					
	Diabetes *n* = 425 *n* fatal cases = 241			Nondiabetes *n* = 6009 *n* fatal cases = 2078		
	HR	95% CI	*p* value	HR	95% CI	*p* value
*Oral health*						
Total number of extractions, *n*	1.01	0.99–1.02	0.245	1.01	**1.01–1.02**	**<0.001**
Total number of tooth extractions by group, test of trend	1.03	0.93–1.14	0.556	1.04	**1.002–1.08**	**0.042**
Total number of tooth extractions by group						
1 = 0-1, reference	1.00	—	—	1.00	—	—
2 = 2–4	1.01	0.73–1.39	0.960	0.84	**0.75–0.93**	**0.010**
3 = 5–8	0.86	0.57–1.30	0.478	0.86	**0.75–0.99**	**0.033**
4 = 9–31	1.14	0.79–1.66	0.477	1.15	**1.001–1.32**	**0.049**
5 = 32	1.23	0.67–2.24	0.502	1.49	**1.19–1.87**	**0.001**
Tooth extraction by infection (TE)	0.93	0.72–1.21	0.58	1.09	0.996–1.187	0.06
Periodontitis (PI)	1.13	0.74–1.71	0.58	0.997	0.824–1.207	0.98
TE and PI combined (OI)	0.92	0.71–1.20	0.53	1.11	**1.02–1.21** ^∗^	**0.02**
*General measurements*						
Age in 2000 years	1.13	**1.09–1.17**	**<0.01**	1.13	**1.12–1.14**	**<0.01**
Glucose nonfasting mmol/l	0.995	0.96–1.03	0.73	1.07	**1.04–1.10**	**<0.01**
Systolic blood pressure mmHg	1.003	0.997–1.01	0.30	1.004	**1.002–1.006**	**<0.01**
BMI Kg/mm^2^	0.998	0.96–1.03	0.91	0.995	0.98–1.01	0.44
Total cholesterol mmol/l	1.07	0.95–1.21	0.26	0.93	**0.89–0.97**	**<0.01**
Triglycerides mmol/l	1.07	0.97–1.17	0.18	0.996	0.95–1.04	0.858
HDL mmol/l	1.16	0.84–1.59	0.38	0.95	0.88–1.06	0.337
hs-CRP mmol/l *n* diabetes 331 *n* nondiabetes 4662	1.02	**1.01–1.03**	**0.01**	1.01	**1.004–1.01**	**<0.01**
hs-CRP mmol/l quartile values						
1 = 0–0.81, reference	1.00	—	—	1.00	—	—
2 = 0.82–1.67	0.66	0.42–1.05	0.078	1.09	0.94–1.27	0.243
3 = 1.68–3.46	0.72	0.45–1.16	0.181	1.27	**1.10–1.47**	**0.001**
4 = 3.47+	1.43	0.94–2.18	0.098	1.84	**1.60–2.11**	**<0.001**
Alcohol drinking 4–7 times per week	0.82	0.62–1.09	0.17	1.10	**1.002–1.20**	**0.045**
Daily smoking Yes	1.25	0.91–1.72	0.17	1.92	**1.74–2.11**	**<0.01**
Level of education 1–12	0.997	0.96–1.03	0.85	0.96	**0.95–0.97**	**<0.01**

^∗^Bold indicates *p* value <0.05.

**Table 3:** Multivariate analyses^∗^ for prediction of total mortality by oral infection variables and hs-CRP in diabetes and nondiabetes. A 12 1/2-year follow-up of Oslo men of the Oslo II study of 2000.

	Tooth extraction any cause	Tooth extraction any cause, group^∗^	Tooth extraction infection (TE)^∗^	Periodontitis (PI)^∗^	Oral infections (OI)^∗^	hs-CRP mmol/l	hs-CRP mmol/l, quartiles^∗^
	HR^∗^ (95% CI)^∗^ *p* value	HR (95% CI) *p* value	HR (95% CI) *p* value	HR (95% CI) *p* value	HR (95% CI) *p* value	HR (95% CI) *p* value	HR (95% CI) *p* value
*Diabetes*							
Oral infection variable or hs-CRP	1.010	1.048	0.923	1.092	0.941	**1.022**	**1.236**
	(0.995–1.025)	(0.940–1.169)	(0.684–1.245)	(0.717–1.664)	(0.693–1.277)	**(1.011–1.034)**	**(1.061–1.439)**
	0.210	0.399	0.600	0.682	0.695	**<0.001**	**0.006**
*Nondiabetes*							
Oral infection variable or hs-CRP	**1.007**	1.014	1.094	1.016	**1.119**	**1.006**	**1.086**
	**(1.001–1.013)**	(0.975–1.055)	(0.991–1.2079)	(0.835–1.237)	**(1.012–1.237)**	**(1.004–1.009)**	**(1.131–1.243)**
	**0.023**	0.488	0.075	0.874	**0.029**	**<0.001**	**<0.001**

^∗^All analyses are adjusted for age, years of education, and daily smoking. 95% CI refers to 95% confidence interval; HR: hazard ratio; OI = TE and/or PI, group = stratified groups of numbers of extractions of any cause: 1 = 0-1, 2 = 2–4, 3 = 5–8, 4 = 9–31, 5 = 32, quartiles = analyses by quartile values of hs-CRP.
